# Challenging the giant: A case report on a huge sacrococcygeal chordoma and its radiological insights

**DOI:** 10.1016/j.radcr.2024.10.157

**Published:** 2024-12-02

**Authors:** Sakshi Dudhe, Devyansh Nimodia, Gaurav V. Mishra, Pratapsingh H. Parihar, Paritosh Bhangale, Anjali Kumari, Rishitha Kotla

**Affiliations:** a Department of Radiodiagnosis, Datta Meghe Institute of Medical Sciences, Sawangi, Wardha, Maharashtra 442001, India; b Department of Psychiatry, Datta Meghe Institute of Medical Sciences, Sawangi, Wardha, Maharashtra 442001, India

**Keywords:** Sacral chordoma, Tumor, Radiology, CT, MRI, USG

## Abstract

Sacral chordoma is a rare osseous tumor of malignant origin. Remnants of the notochord in the region of sacrum and coccyx is said to be the origin of these tumors. Patients generally have delayed presentation, which is responsible for larger tumor size. Early detection of sacral chordoma is therefore very important. We present a clinical case on a 56-year-old male patient who presented with chronic lower back pain for 3 years, predominantly in the sacral region. He also had occasional bouts of constipation. Diagnostic imaging modalities used encompass ultrasonography, magnetic resonance imaging, and computed tomography scans. Radiological imaging of this patient revealed a giant mass within the pelvis. The mass was involving the lower sacrum and coccyx region predominantly. Associated local osteolytic changes were also noted, which are a characteristic feature of sacral chordoma. The patient underwent surgical excision of the tumor. Subsequent histopathological analysis gave the diagnosis of chordoma of sacral region.

Sacral chordoma should be a consideration in differential diagnosis whenever the patient presents with persistent chronic lumbosacral pain, discomfort, and neurological impairments. Surgical excision followed by radiotherapy is useful in improving clinical outcomes for individuals diagnosed with sacral chordoma. This enhances overall patient prognosis and reduces the risk of local recurrence. The diagnosis of sacral chordoma is a challenge for diagnostic radiologists. Quicker identification, assertive surgical intervention, and suitable adjuvant therapies for chordoma are essential for enhancing patient outcomes and effectively managing this uncommon malignancy called chordoma.

## Introduction

Chordomas are rare malignant tumors, arising from embryonic remains of primitive notochord. Chordomas constitute almost 1%-4% of all primary bone tumors [1]. The incidence rate of about 0.08 per 100,000 has been noted. The most commonly affected parts include axial skeleton, the mobile spine and the sacrum [2]. Midline or paramedian location is noted commonly. About 50%-60% of cases involve the sacrococcygeal region. Patients mostly present in the age distribution of fourth to seventh decade. Peak presentation of this tumor is noted in 5th decade. Males are more commonly affected than female. Male to female ratio is approximately 2:1. Five-year survival rate of about 50% and 10-year survival rate of about 30% is noted in these cases. Sacral chordoma is a very slow growing tumor and hence it is commonly larger in size at presentation [5]. Symptoms of patients can include back pain, weakness, constipation, numbness, and incontinence [3].

Imaging modalities used for diagnosis of sacral chordomas are plain radiography, computed tomography (CT), and magnetic resonance (MR) imaging. Destruction of the involved part of sacrum can be known on plain radiographs. But CT and MRI have been frontiers for assessing the soft tissue extension of the tumor and for proper evaluation of the sacrococcygeal chordoma [4]. Surgery treatment is the mainstay of this tumor. Radiation therapy with gross excision of the tumor mass is the current standard treatment that is advocated for improving the patients long term survival of these patients [3].

## Case presentation

A 56-year-old male patient presented to the orthopedics department with complaints of chronic lower back pain for 3 years, predominantly in the sacral region. The lower back pain worsened on sitting and was relieved on standing. Pain had exacerbated recently, and hence decided to visit the hospital. He also complained of lower limb weakness for 3 months. Patient gave a history of occasional bouts of constipation and abdominal cramps. He was hypertensive and a known case of chronic renal failure. On evaluation, the general and examination was within normal limits. The local examination revealed tenderness at the sacral region.

**Fig. 1:**
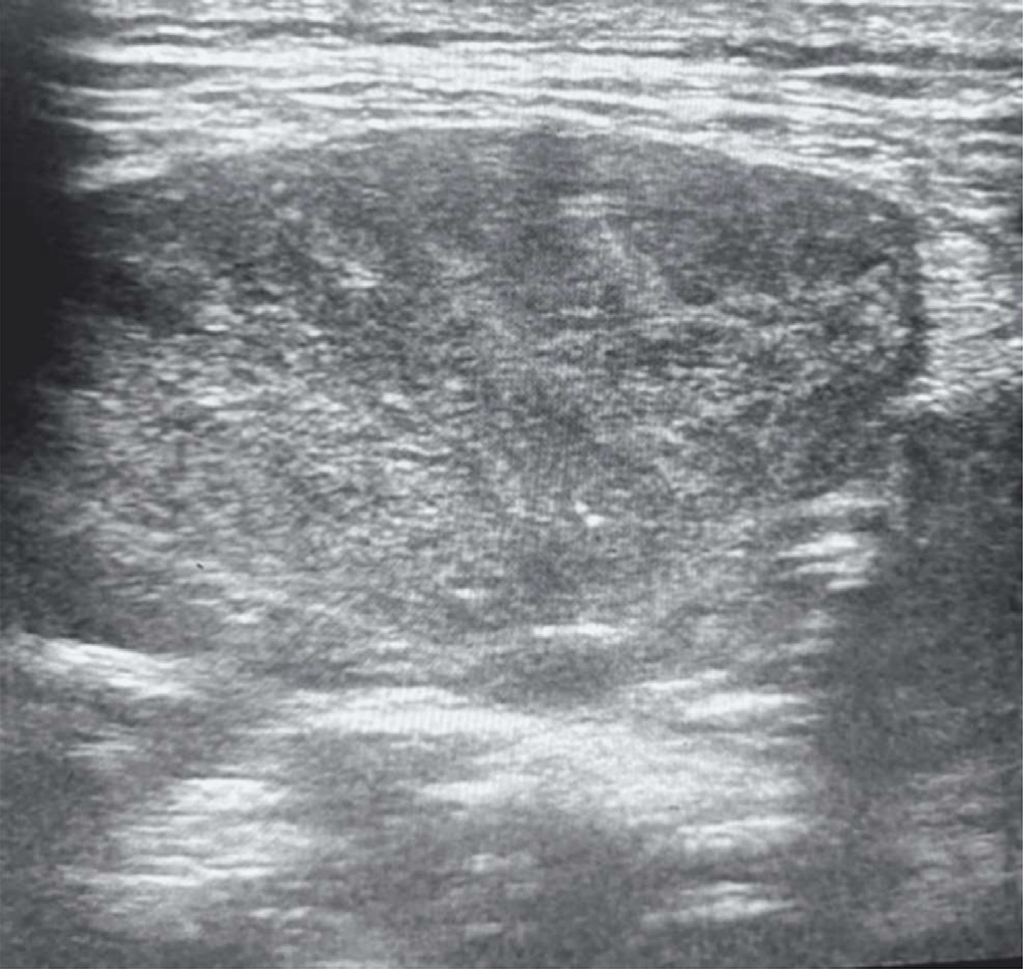
Ultrasound grey scale B mode-showing large mass lesion in the sacral region which appears heterogeneously hypoechoic with few areas of calcifications within.

**Fig. 2:**
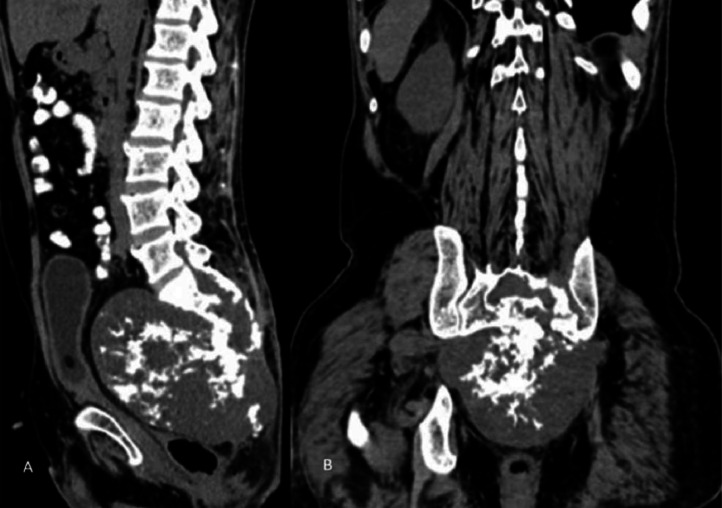
CT sagittal (A) and coronal (B) sections showing large well circumscribed expansile soft tissue density mass lesion with intralesional calcifications noted in the pelvis in the region of lower sacrum and coccyx causing its destruction.

**Fig. 3:**
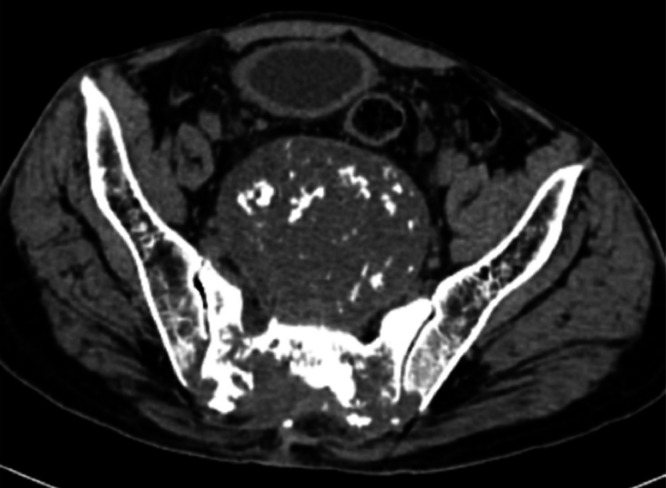
CT axial soft tissue window showing lesion involving presacral fat and displacing & compressing the rectum anteriorly and reaching upto subcutaneous region posteriorly, involving medial part of gluteal muscles.

**Fig. 4:**
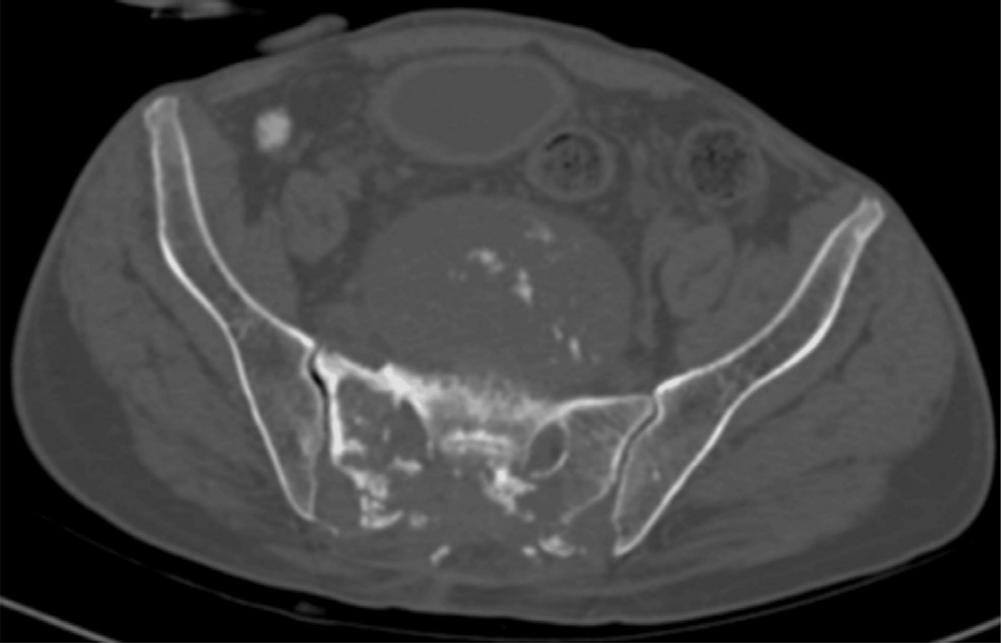
CT axial section bone window showing destruction of sacrum caused by the lesion.

A plain radiograph was advised, which showed a large, rounded radiolucent mass lesion with multiple calcifications in the pelvis with destruction of the lower sacral vertebrae and coccyx. Ultrasound of the sacral region was also done, which revealed a large heterogeneously hypoechoic mass lesion in the sacral region with few areas of calcifications as shown in Fig. 1. For deeper extension and involvement CT scan and MRI were advised. On contrast enhanced CT scan large nonenhancing well circumscribed lytic expansile soft tissue density mass lesion with intralesional calcifications was noted in the pelvis, in the region of lower sacrum and coccyx as shown in Fig. 2. The large mass lesion measured 16 × 13 × 13.8 cm in dimensions. Anteriorly, the mass lesion was involving presacral fat and displacing & compressing the rectum. Posteriorly, it was reaching up to subcutaneous region, involving medial part of gluteal muscles as shown in Fig. 3. Destructive lysis of lower sacral vertebrae and coccyx was noted on CT bone window as shown in Fig. 4.

**Fig. 5:**
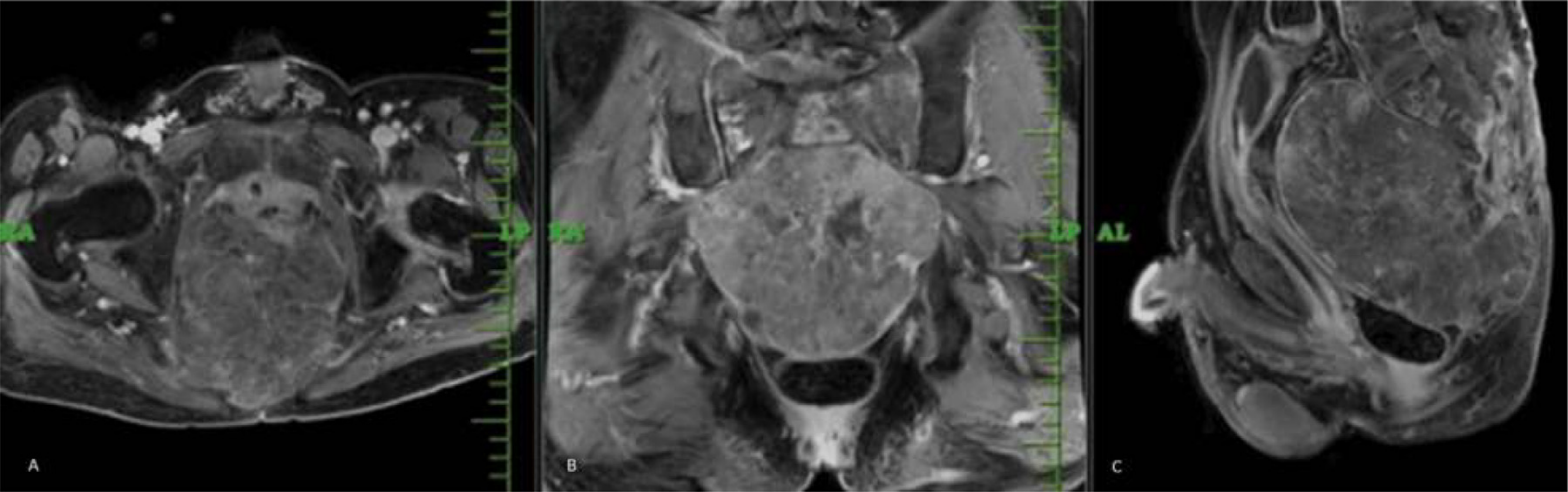
MRI post contrast axial(A), coronal (B), sagittal (C) sections showing large heterogenously enhancing mass lesion involving lower sacral vertebra and coccyx.

**Fig. 6:**
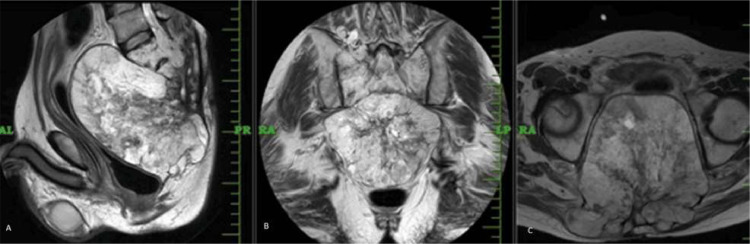
MRI T2 weighted sagittal (A), coronal (B), axial (C) sections showing heterogeneously hyperintense mass lesion in lower sacral and coccyx region.

For studying superior soft tissue extension, contrast MRI was advised. It revealed large heterogeneously enhancing mass lesion in sacrum and coccyx, appearing hypointense on T1 weighted sequences along with heterogeneously hyperintense signal on T2 weighted imaging as shown in Fig. 5, Fig. 6. The mass lesion was seen to have intralesional calcifications and few foci of hemorrhage within. The patient underwent gross total resection of the tumor along with partial sacrectomy and coccygectomy. Adjuvant radiation therapy was started for the patient. Patient is now doing well, relieved of major concerning symptoms. He has been advised 2 monthly follow up.

## Discussion

Chordomas represent rare neoplasms, constituting approximately 2%-4% of all primary malignant osseous tumors. These tumors generally emerge from the embryonic remnants of the notochord, manifesting at various locations along the neural axis. The localization of these tumors within the axial skeleton, in descending order of frequency, is predominantly observed in the sacrococcygeal region (50%), followed by the spheno-occipital region (30%-50%), and other spinal segments (15%) [4]. The sacral region is markedly the most prevalent site for the occurrence of this tumor, representing 50% of all chordomas [5]. The neoplasm exhibits a slow growth pattern; consequently, there is often a protracted interval of several months to a few years between the onset of clinical symptoms and the establishment of a definitive diagnosis [6]. Most common symptoms include back pain, paresthesia, constipation, feeling of weakness and urge incontinence [3]. The classical characteristics of sacrococcygeal chordoma are identified as a soft tissue mass that exhibits aggressive osseous destruction, alongside the invasion of adjacent soft tissues and neurovascular structures within the pelvic cavity [7].

Sacrococcygeal chordoma is characterized by the presence of midline or paramedian osteolytic changes that extend to the inferior regions of the sacrum and coccyx, typically accompanied by an anterior soft tissue mass. The osteolytic lesions exhibit well-defined borders and may present with cortical bulging in many cases. Calcifications are common and are observed in approximately 50% to 60% cases of sacral chordomas. Computed tomography (CT) can be done to know the extent of osseous involvement including sacroiliac joint, spinal canal invasion, the exact size of the lesion and mass effect on adjacent structures. Calcifications, cortical bulging, and osteolytic areas are seen in these cases. Good postcontrast enhancement within the tumor is noted. On MRI, chordomas typically present with low signal on T1-weighted sequences, coupled with moderate postcontrast enhancement after gadolinium administration. On T2-weighted sequences, the neoplasm exhibits an exceedingly elevated signal intensity [8].

In MRI, the most prominent characteristic of a chordoma is the pronounced high signal on T2-weighted images, though it is nonspecific. There should be a notable existence of lobulated sacral mass with areas of hemorrhage and calcification, which will point out the diagnosis of chordoma. The differential diagnosis concerning sacral tumors associated with osseous destruction encompasses chordoma, chondrosarcoma, giant cell tumor, myxopapillary ependymoma, plasmacytoma, and metastatic lesions. The giant cell tumor ranks as the second most prevalent primary neoplasm of the sacrum subsequent to chordoma [3].

In the differential diagnosis of an expansile lesion within the sacrum, the primary conditions to consider include giant cell tumor, chondrosarcoma, ependymoma, plasmacytoma, and a solitary metastatic lesion [7]. The prognosis for cranial chordoma is regarded as unfavorable. The average survival duration following the onset of clinical symptoms is approximately 4-5 years, notwithstanding surgical intervention and/or radiotherapy. Conversely, the prognosis for sacrococcygeal chordoma is comparatively more favorable, with an average survival duration ranging from 8 to 10 years. Radiotherapy may elicit substantial regression of cranial and spinal tumors, with remission periods extending from 1 to several years. A synergistic approach involving radiotherapy and surgical intervention aimed at decompressing vital structures is perceived as offering the most promising opportunity for prolonging survival [6].

Treatment involves radical surgical resection, which ensures the establishment of tumor-free margins surrounding the neoplasm to prevent the effraction of the tumor contents; the operative challenges predominantly stem from the volume of the tumor. The genitourinary tract involvement can proceed to motor and sensory symptoms. Radiotherapy given for cases of partially resected or inoperable tumors has palliative and analgesic effects. Post curettage local recurrences are observed depending upon the location or size of the tumor in 80% of cases. Hematogenous metastases are rare and observed chronic disease progression [8].

Partial resection and radiation therapy are indicated treatment for sacral chordomas; however, it does not change the long-term management of this tumor. For efficient management of sacrococcygeal chordoma, early diagnosis has its own importance. This will enable the patient to undergo surgical removal of the tumor. Follow up of the patient is important [9]. Intratumoral chemotherapy and surgical intervention has been advocated in these tumors [10].

## Conclusion

This case report throws light on the clinical challenges and management strategies associated with a huge sacrococcygeal chordoma. The patient should be evaluated for significant clinical symptoms in correlation to tumor size and location. Early prompt diagnosis and the significance of advanced imaging modalities in planning surgical strategies is of utmost importance. Surgery ensures favorable prognosis and better disease management. This case provides invaluable insights into the management of intricate sacrococcygeal neoplasms and reinforces the imperative for continuous exploration aimed at optimizing therapeutic protocols and enhancing the overall quality of life for the patients.

## Patient consent

Informed and written consent was obtained from the patient.
